# Bone marrow mesenchymal stem cell‐derived extracellular vesicles promote corneal epithelial repair and suppress apoptosis via modulation of Caspase‐3 *in vitro*

**DOI:** 10.1002/2211-5463.13804

**Published:** 2024-04-29

**Authors:** Vasudeva Tati, Sreya Mitra, Sayan Basu, Sachin Shukla

**Affiliations:** ^1^ Prof. Brien Holden Eye Research Centre, Hyderabad Eye Research Foundation L V Prasad Eye Institute Hyderabad India; ^2^ Sudhakar and Sreekanth Ravi Stem Cell Biology Laboratory, Centre for Ocular Regeneration L V Prasad Eye Institute Hyderabad India; ^3^ Shantilal Shanghvi Cornea Institute, L V Prasad Eye Institute Hyderabad India

**Keywords:** apoptosis, bone marrow, Caspase‐3, corneal injury, extracellular vesicles, mesenchymal stem cells

## Abstract

Corneal injuries are the major cause of blindness and visual impairment. Available treatments are limited by their efficacy and side effects. Mesenchymal stem cell‐derived extracellular vesicles are presumed as functional equivalents and potential candidates for cell‐free therapy. This study reports isolation and characterization of extracellular vesicles from human bone marrow mesenchymal stem cells and evaluates their role in mediating epithelial repair and apoptosis in cultured corneal epithelial cells through scratch assay, PCR, immunofluorescence, and flow cytometry *in vitro*. The isolated extracellular vesicles were spherical, < 150 nm in diameter, and characterized as CD9^+^, CD63^+^, CD81^+^, TSG101^+,^ and Calnexin^−^. Further, these vesicles promoted corneal epithelial repair by enhancing proliferation and suppressed apoptosis by regulating the expression of *BAD*, *P53*, *BCL‐2*, and cleaved CASPASE‐3*.* Thus, our results suggest that BM‐MSC‐EVs might have the potential to be used for the treatment of injury‐induced corneal epithelial defects. Clinical translation of this work would require further investigations.

## Graphical abstract

We investigated the impact of BM‐MSC‐EVs on corneal epithelial repair and apoptosis using *in vitro* wound healing and apoptotic assays. Our findings suggest that BM‐MSC‐EVs promote corneal epithelial repair by enhancing proliferation (Ki67) and reduce apoptosis in human corneal epithelial cells by modulating the expression of Caspase‐3 (Image created with https://www.biorender.com/).

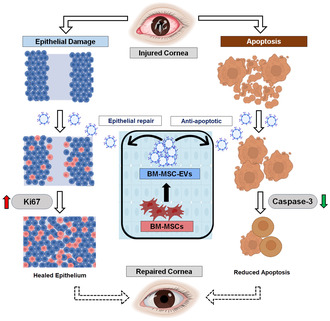

**Abbreviations**
ADSC‐EVs: adipose stem cell‐derived extracellular vesicles
ANOVA: analysis of variance
ATCC: American Type Culture Collection
BAD: Bcl‐2‐associated death promoter
BAK: Benzalkonium chloride
BCL‐2: B‐cell lymphoma 2
bFGF: basic fibroblast growth factor
BM‐MSCs: bone marrow‐derived mesenchymal stem cells
BSA: bovine serum albumin
CASPASE: cysteine‐aspartic proteases
CCL: chemokine ligand
CD: cluster of differentiation
cDNA: complementary deoxyribonucleic acid
DAPI: 4,6‐diamidino‐2‐phenylindole
DMEM F12: Dulbecco's modified Eagle's medium, nutrient mixture F‐12
ECM: extracellular matrix
EGF: epidermal growth factor
EVs: extracellular vesicles
FBS: fetal bovine serum
FITC: fluorescein isothiocyanate
GMP: good manufacturing practices
HCECs: human corneal epithelial cells
HGF: hepatocyte growth factor
HRP: horseradish peroxidase
HUMSC‐sEVs: human umbilical cord mesenchymal stem cell‐derived small extracellular vesicles
IDV: integrated density value
IFN‐γ: interferon gamma
IgG: immunoglobulin G
IL‐10: interleukin‐10
iMSCs: induced pluripotent stem cell‐derived mesenchymal stromal cells
iPSC: induced pluripotent stem cell
lncRNAs: long non‐coding RNAs
miRNA: micro RNA
MISEV: minimal information for studies of extracellular vesicles
mRNA: messenger RNA
MSC: mesenchymal stem cell
MSCBM: mesenchymal stem cell basal medium
MSC‐EVs: mesenchymal stem cell‐derived extracellular vesicles
NFκB: nuclear factor kappa‐light‐chain‐enhancer of activated B cells
NTA: nanoparticle tracking analysis
PBS: phosphate‐buffered saline
PCR: polymerase chain reaction
PDGF: platelet‐derived growth factor
PI: propidium iodide
PTEN/PI3K/Akt: phosphatase and tensin homolog/phosphatidylinositol 3‐kinase/protein kinase B
PVDF: polyvinylidene fluoride
RAP 1: Ras‐related protein 1
RIPA: radioimmunoprecipitation assay
RNA: ribonucleic acid
RT‐PCR: reverse transcriptase polymerase chain reaction
SDS/PAGE: sodium dodecyl sulfate‐polyacrylamide gel electrophoresis
SEM: Scanning Electron Microscopy
STS: Staurosporine
TBS‐T: Tris‐buffered saline‐Tween‐20
TEM: Transmission Electron Microscopy
TGF‐β: transforming growth factor beta
TNF‐α: tumor necrosis factor‐alpha
TSG101: tumor susceptibility gene 101
VEGF: vascular endothelial growth factor
Z‐DEVD‐FMK: Z‐Asp‐Glu‐Val‐Asp‐Fluoromethylketone

The cornea is one of the distinctive tissues as it provides structural integrity to the eye. Any damage to its constitutive layers including corneal epithelium may compromise vision and lead to blindness [1, 2]. Persistent corneal epithelial defects caused by traumatic injuries, thermal and chemical burns [3], ocular surface inflammatory disorders [4, 5] and surgical interventions, are the result of re‐epithelialization failure. An injured cornea needs a robust healing process involving migration, proliferation, and differentiation of corneal epithelial cells.

Despite many effective treatments, mesenchymal stem cell (MSC)‐based therapies are one of the most widely accepted cell‐based therapies due to innate multipotent differentiation potential, pro‐angiogenic, anti‐apoptotic, and immunomodulatory properties of MSCs [6, 7]. Among the various non‐ocular tissue sources, bone marrow‐derived MSCs (BM‐MSCs) remain the primary choice for clinical trials due to their proven safety, tolerance, and efficacy [8, 9, 10, 11, 12, 13, 14]. Paracrine secretions of MSCs, collectively called secretome, include growth factors, extracellular vesicles (EVs), cytokines, chemokines, angiogenic factors, anti‐apoptotic factors, and extracellular matrix (ECM) constituents and are primarily responsible for MSC‐based therapeutic properties [15, 16, 17, 18, 19].

Mesenchymal stem cell‐derived EVs (MSC‐EVs) are presumed to be the functional equivalents of MSCs and act by paracrine mechanisms. These EVs are membrane‐bound small vesicles, that range from 30 to 150 nm in size and contain various biomolecules which are involved in diverse functions like signaling, fusion, and endocytosis [20]. MSC‐EVs are proposed to have several advantages over conventional MSCs for treating corneal injuries being safer, biocompatible, and relatively less immunogenic. These can be stored and transported more easily due to their encapsulation capability [21]. MSC‐EVs have also been shown to reduce corneal scarring and restore corneal transparency after injuries [22]. In corneal wound healing, MSC secretome function by improvising cell viability, proliferation, and ECM formation [23]. BM‐MSC‐derived exosomes have shown therapeutic benefits in retinal ganglionic cells, similar to MSCs [24]. However, the characterization of isolated MSC‐EVs given in the widely accepted minimal information for studies of extracellular vesicles (MISEV) 2018 guidelines [25] which are recently updated to MISEV2023 [26] and their functional role in corneal epithelial repair and, in particular, apoptosis is poorly studied. This study aims to further investigate the role of BM‐MSC‐EVs in corneal epithelial repair and apoptosis using cultured human corneal epithelial cells (HCECs) *in vitro*.

## Materials and methods

### Ethical approval

This study was approved by Institutional ethical committees (LEC‐BHR‐P12‐20‐559 and LVPEI‐ IC‐SCR‐05‐21‐007).

### Cell culture

Human BM‐MSCs (#PT‐2501, Tissue acquisition number# 35671, Batch number# 18TL113327, isolated from the bone marrow of 31 years old male) were procured from Lonza Bioscience, Walkersville, MD, USA, and maintained in MSC basal media (MSCBM, #PT‐3238) with growth supplement (#PT‐4106E), L‐Glutamine (#PT‐4107E), and Gentamicin sulfate‐Amphotericin B (#PT‐4504E), as per the manufacturer's instructions. HCECs were procured from ATCC, USA (#HCE‐2 [50. B1] CRL‐11135, isolated from the cornea of a Black male donor, deposited by CR Kahn, Gillette Medical Evaluation Laboratories, USA, cited in US Pat. No.5672498). These were cultured in complete DMEM F12 media [Dulbecco's modified Eagle's medium, Nutrient mixture F‐12 (#10565–018, Gibco, Life Technologies Corporation, New York, NY, USA) supplemented with 10% Fetal bovine serum (FBS) (#RM9952, Hi‐Media, Mumbai, India), Penicillin (100 units·mL^−1^), Streptomycin (100 μg·mL^−1^), and Amphotericin B (0.25 μg·mL^−1^) (#A002, Hi‐Media, Mumbai, Maharashtra, India)] as per the supplier's instructions. The cells were maintained at a temperature of 37 °C and a CO_2_ concentration of 5% in a controlled environment. Both the hBM‐MSCs and HCECs were used at passage#3 (P#3) in this study.

### Isolation and quantification of BM‐MSC‐EVs

The EVs were isolated from BM‐MSCs and quantified using Total Exosome Isolation Kit (#4478359, Invitrogen, Waltham, MA, USA) and EXOCET (#EXOCET96A‐1, System Biosciences, Palo Alto, CA, USA) kit, respectively, as per the manufacturer's instructions. Briefly, at 90% confluency, the spent medium was discarded and BM‐MSCs were grown in DMEM‐F12 medium supplemented with exosome‐depleted FBS (#A2720801, Thermo Scientific, Waltham, MA, USA) for 24 h. The EVs were isolated from the conditioned medium, resuspended in filtered 1× Phosphate‐buffered saline (PBS) (#2120600, Gibco, Carlsbad, CA, USA) and stored at −80 °C, till further use.

For quantification, the lysis buffer supplied in the kit and EV samples were incubated in a 4 : 1 ratio at 37 °C for 5 min and centrifuged at 1500 ***g*** for 5 min, resulting supernatant was transferred to a new tube and placed on ice. In a 96‐well plate, reaction buffer was added followed by kit standards and EV samples to respective wells, which was incubated for 20 min. The absorbance was recorded at 405 nm using a UV spectrophotometer (SpectraMax M3, Molecular Devices, San Jose, CA, USA) and a standard curve was plotted.

### Characterization of BM‐MSC‐EVs

#### Transmission electron microscopy (TEM)

For sample preparation, 1 × 10^8^ BM‐MSC‐EVs and 2% Glutaraldehyde (#46080‐L05, SD Fine‐Chem Limited, Mumbai, Maharashtra, India) were mixed (1 : 1, v/v) and incubated for 30 min for fixation. Subsequently, 6 μL of the fixed sample was applied to the carbon‐coated copper grids. The latter were washed with double‐distilled water and excess water was removed with blotting paper. Negative staining was performed with 6 μL of 1.5% uranyl acetate (#81405, Sisco Research Laboratories, Mumbai, Maharashtra, India) for 30 s and the grids were placed under an incandescent bulb for 2 h for expedited drying following which the grid was left for air drying for 2 days. Images were captured using a transmission electron microscope (JEOL 2100 Plus, Akishima, Japan) operating at 120 kV.

#### Scanning electron microscopy (SEM)

For sample preparation, 1 × 10^8^ BM‐MSC‐EVs were placed on coverslips, air dried, and fixed with 2.5% Glutaraldehyde (#46080 L05, SD Fine‐Chem Limited) for 2 h. Subsequently, samples were dehydrated using upgrade concentrations of Ethanol (10%, 30%, 50%, 70%, 90%, and 100%, #2005786, Hayman, Witham, UK), dried overnight, and sputtering was done. Images were captured using a scanning electron microscope (EVO 18, ZEISS, Oberkochen, Germany).

#### Nanoparticle tracking analysis (NTA)

The size and concentration of EVs, based on Brownian motion, were measured by NanoSight NS300 (Malvern Instruments Ltd., Worcestershire, UK) according to the manufacturer's instructions. Briefly, all samples were diluted in water at a ratio of 1 : 500. For each measurement, three videos of 10‐ and 60‐s duration were recorded and analyzed using the NanoSight software NTA 3.4 Build 3.4.003, with a detection threshold of 10.

#### Western blotting

Isolated EVs were suspended in RIPA lysis buffer (# TCL131, Hi‐Media) (1 : 1, v/v) and subjected to 10–15% SDS/PAGE. Proteins were blotted on a PVDF membrane (#sc‐3723, Santa Cruz Biotechnology, Inc., Dallas, TX, USA) using semi‐dry transfer method. Blocking was done using 2% Bovine serum albumin (BSA, # MB‐083, Hi‐Media) in 1× Tris‐buffered saline with 0.01% Tween‐20 (1xTBS‐T) for 1 h. Subsequently, membranes were probed overnight with respective primary antibodies (1 : 500 each, Invitrogen): anti‐CD63 (#10628D), ‐CD81 (#MA5‐13548), ‐TSG101 (#MA1‐23296), and ‐Calnexin (#MA3‐027), following incubation with species‐specific secondary antibody (1 : 3000, anti‐mouse HRP conjugate, #HPO6; B Genei Laboratories Pvt. Ltd., Bangalore, Karnataka, India) for 1 h. All antibodies were diluted in 1xTBS‐T containing 2% BSA and membranes were washed with 1xTBS‐T (4 times for 15 min each). The blots were developed using FemtoLUCENT plus‐HRP (#786003, G‐BIOSCIENCES, St. Louis, MO, USA) on X‐ray sheets under dark conditions.

#### Immunofluorescence

Briefly, 1 × 10^8^ EVs were dried on 8‐well glass bottom μ‐Slide (#80827, ibidi GmbH, Gräfelfing, Germany), washed with PBS, and fixed with 4% paraformaldehyde (#TC703‐500g; Himedia Laboratories Pvt. Ltd., Mumbai, Maharashtra, India). Further fixation and blocking were performed using H_2_O_2_: Methanol (4 : 1, v/v) and 2% BSA (in 1xTBS‐T), respectively. EVs were stained overnight with respective primary antibodies (anti‐CD9, #10626D; ‐CD63, #10628D; ‐TSG101, #MA1‐23296, ‐Calnexin, #MA3‐027; Invitrogen), following incubation with a secondary antibody (goat anti‐mouse IgG‐FITC, #SF131, GeNei™, Bangalore, India) for 1 h and mounted with Fluoroshield™ with DAPI (#F6057, Sigma, Burlington, MA, USA). The slides were observed under the fluorescence microscope (Axio Scope A1, Carl Zeiss, Oberkochen, Baden‐Württemberg, Germany).

### *In vitro* wound healing assay

The effect of BM‐MSC‐EVs on corneal epithelial repair was evaluated through *in vitro* wound healing assay [20, 27, 28]. HCECs were cultured in 12 well plates until 100% confluency. A vertical scratch was made across the diameter of the well using a 200 μL tip and BM‐MSC‐EVs (1 × 10^8^) were added. HCECs grown in DMEM‐F12 were used as a mock. Healing was observed at different time intervals (0, 12, 24, and 36 h) under the microscope (Primovert microscope, Zeiss) and images were analyzed using imagej software (version: 64‐bit, Java 1.8.0_172) (https://imagej.nih.gov/ij/). To determine whether the healing was primarily driven by cell proliferation or migration, HCECs were treated with Mitomycin C (10 μg·mL^−1^) for 3 h before scratch and followed as mentioned above. BM‐MSC‐EVs (1 × 10^8^) were added to both untreated and Mitomycin C‐treated scratched HCECs.

To evaluate the direct impact of MSC‐EVs on proliferation of HCECs during wound healing, proliferation assay was performed using Ki67 staining (anti‐Ki‐67 monoclonal antibody, #Ab16667, Abcam, Waltham, MA, USA). The mean fluorescence intensity of the Ki67‐positive cells was quantified using imagej software and expressed as arbitrary units (a.u).

### Apoptosis

The apoptosis was experimentally induced in HCECs with H_2_O_2_ (#18706, Fisher Scientific Ltd., Mumbai, India; 200 μmol·L^−1^ for 4 h). Subsequently, the test samples were incubated with/without BM‐MSC‐EVs (1 × 10^8^) for 24 h in DMEM‐F12. Untreated HCECs were used as a control. Induction of HCECs with Staurosporine (STS, # sc‐3510, Santa Cruz Biotechnology, Inc., 0.25 μM for 3 h [29]), and Benzalkonium chloride (BAK, #43648 was procured from Sisco Research Laboratories; 0.005% (v/v) for 15 min [30, 31]) was also used for validation of apoptosis.

Pre‐treatment of HCECs with a Caspase‐3 inhibitor: Z‐DEVD‐FMK (#sc‐3075, Santa Cruz Biotechnology, Inc.; 10 μM for 1 h [32]) was used to evaluate the Caspase‐3 dependent modulation of apoptosis by BM‐MSC‐EVs.

#### RNA isolation and PCR

The mRNA expression of candidate apoptotic genes (*BAD*, *P53*, *BCL‐2*, and *CASPASE‐3*) was quantified by semi‐quantitative and quantitative RT‐PCR (40 cycles each) using gene‐specific primers (Table S1). RNA was isolated using RNeasy mini kit (#74104, QIAGEN, Hilden, Germany) and quantified using NanoDrop 2000c Spectrophotometer (ThermoFisher Scientific, Waltham, MA, USA). The cDNA was synthesized using a Superscript IV first‐strand synthesis system (#18090050, ThermoFisher Scientific) as per the manufacturer's instructions. Quantitative real‐time PCR (7900HT Fast Real‐Time PCR system, Applied Biosystems, ThermoFisher Scientific) was performed using PowerUp™ SYBR green master mix (#A25742, Applied Biosystems, ThermoFisher Scientific). Thermal cycling conditions were as follows: 95 °C for 2 min, followed by 95 °C for 25 s for denaturation, and finally 60 °C for 1 min for annealing/extension. The conditions were kept constant for 40 cycles and dissociation curves were examined. Absolute quantification of gene expression was measured, and *β‐ACTIN* was used as the internal control. Results of semi‐quantitative PCR were quantified in terms of Integrated density values (IDV) using ImageJ software.

#### Cleaved Caspase‐3 antibody

To study the Caspase‐3 mediated regulation of apoptosis, an antibody specific to cleaved Caspase‐3 (Asp175) (#9661, Cell Signaling Technology, Inc., Danvers, MA, USA) was used, as per the manufacturer's instructions. Cleavage of Caspase‐3 indicates its activation which requires preoteolytic processing of its inactive zymogen into activated p17 and p12 fragments. This antibody detects the endogenous levels of large fragment (17/19 kDa) of activated Caspase‐3 resulting from cleavage adjacent to Aspartic acid residue at the P1 position [33, 34].

#### Flow cytometry and immunofluorescence

Single cell suspensions of HCECs from different treatment groups (H_2_O_2_, H_2_O_2_ + BM‐MSC‐EVs, Inhibitor (Z‐DEVD‐FMK), Inhibitor + H_2_O_2_, Inhibitor + H_2_O_2_ + EVs), as applicable, were made and a total of 1 × 10^6^ cells were processed using a Dead cell apoptosis kit with Annexin V (#V13242, Invitrogen) and a Cleaved Caspase‐3 (Asp175) antibody (#9661, Cell Signaling Technology, Inc.), as per the manufacturer's instructions. Data acquisition was done using CytoFLEX Flow Cytometer [Beckman Coulter, Inc., Brea, CA, USA; Excitation at 488 nm and detection at 530 ± 20 nm (Annexin V‐FITC) and above 600 nm (Propidium Iodide, PI)] and analyzed using cytexpert 2.3 software (Beckman Coulter). For immunofluorescence‐based‐detection of cleaved Caspase‐3, HCECs from different treatment groups were fixed and blocking was performed as mentioned above. HCECs were incubated with anti‐cleaved caspase‐3 (Asp‐175) antibody (# 9661S, Cell Signaling Technology, Inc.) overnight, followed by washing with 1xTBS‐T, incubation with Goat anti‐Rabbit IgG Rhodamine conjugated secondary antibody for 1 h, washing with 1xTBS‐T, and mounting with Fluoroshield™ with DAPI (#F6057, Sigma). Anti‐β‐actin antibody (# sc‐47 778, Santa Cruz Biotechnology, Inc.) was used in double immunostaining. The slides were analyzed using the confocal microscope (LSM 880, Carl Zeiss, Oberkochen, Baden‐Württemberg, Germany).

### Statistical analysis

The experiments were performed at least three times (*N* = 3). The data are expressed as the mean ± standard deviation (SD) of the three independent sets of experiments in triplicate. Statistical analysis was performed using Microsoft Excel with one‐way analysis of variance (ANOVA) to compare the means of different groups. A *P*‐value of < 0.05 was considered statistically significant.

## Results

### Characterization of BM‐MSC‐EVs

**Fig. 1:**
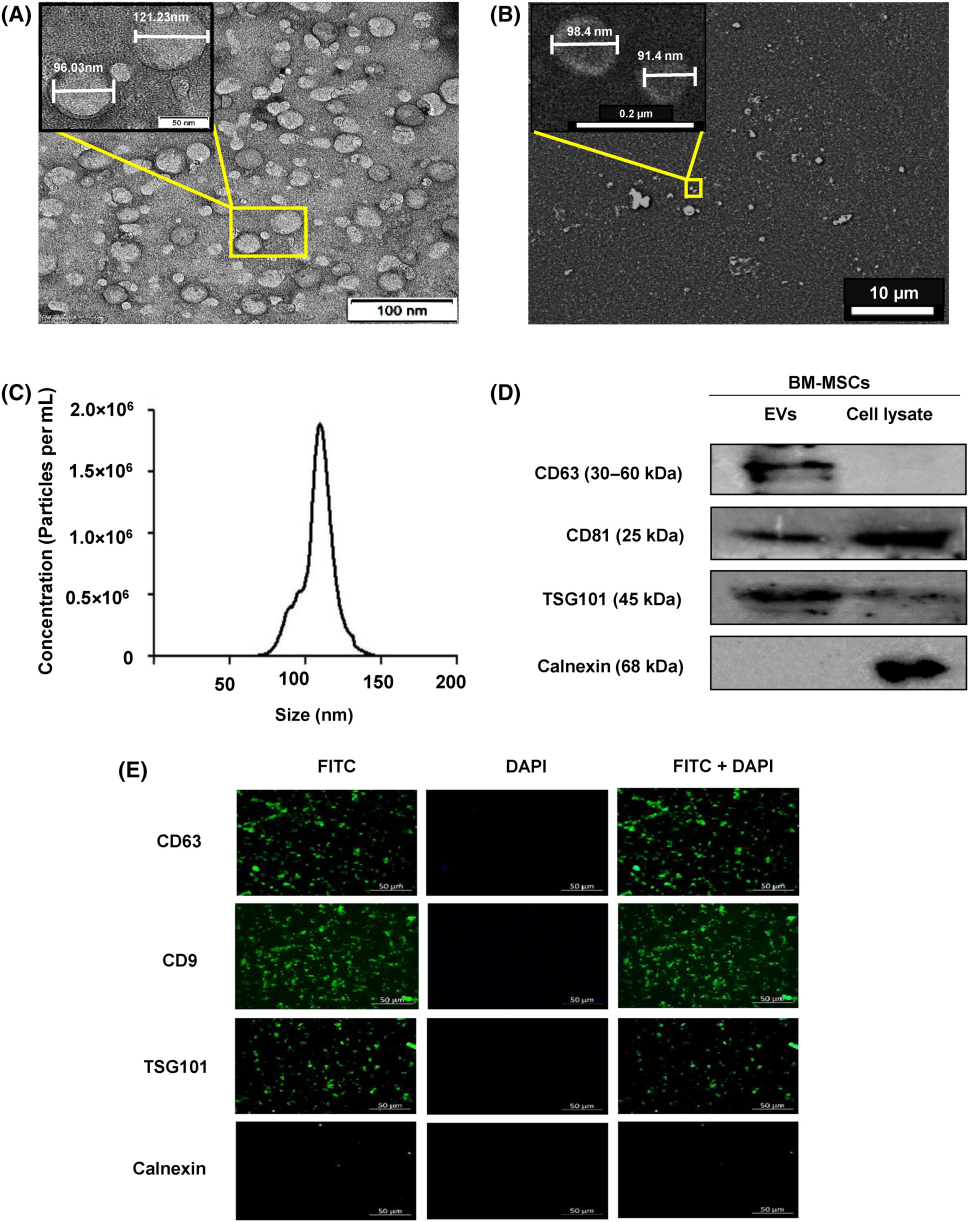
Characterization of BM‐MSC‐EVs. The morphology of isolated BM‐MSC‐EVs was determined by (A) TEM and (B) SEM. The EVs were circular in size with a diameter < 150 nm. (C) NTA results showed EVs with an average size of ~ 120 nm. (D) Western blotting and (E) Immunofluorescence results of BM‐MSC‐EVs showed positive expression of Tetraspanins (CD63, CD9, and CD81), and cytoplasmic marker TSG101, whereas expression of Endoplasmic Reticulum related protein (Calnexin) could not be detected. The cell lysate of BM‐MSCs was used as a control in western blotting. Experiments were performed at least three times (*N* = 3) and representative images are shown.

TEM and SEM analyses of the EVs displayed a uniform circular shape with consistent size distribution (< 150 nm) (Fig. 1A,B) whereas NTA further confirmed the presence of EVs within the range of 50–150 nm with an average of 127 ± 94 nm (Fig. 1C). The quantification of EV standards revealed a highly favorable linear relationship (*R* ^2^ = 0.99), and the EV samples contained 149 × 10^7^ ± 4.94 EVs per 100 μL. Furthermore, the expression of specific protein markers in the EVs was investigated using western blotting and immunostaining. The results demonstrated the expression of tetraspanins (CD63, CD9, and CD81) and cytosolic marker (TSG101), whereas Calnexin could not be detected (Fig. 1D,E).

### Corneal epithelial repair

**Fig. 2:**
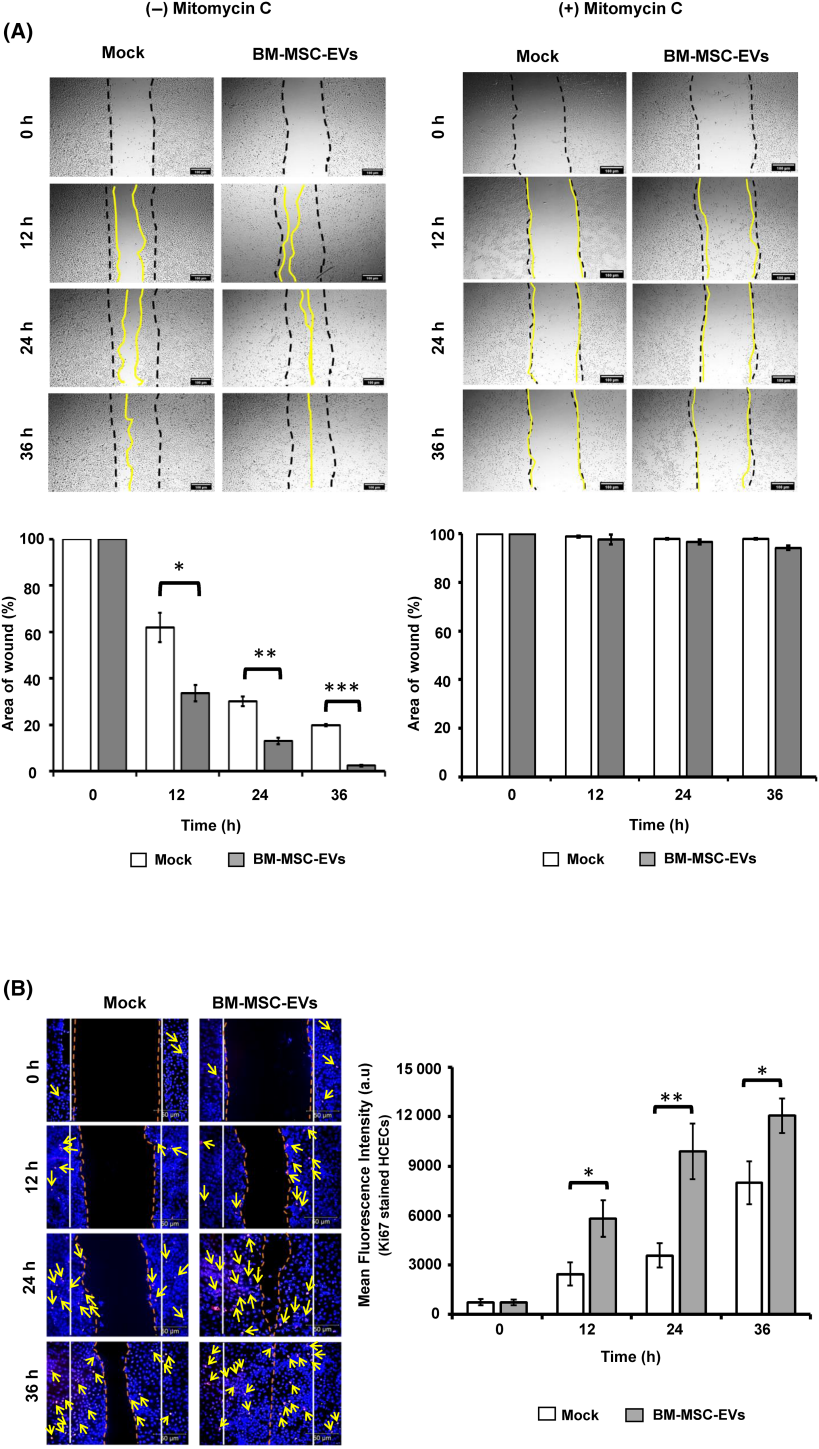
Corneal epithelial repair by BM‐MSC‐EVs. (A) Scratch assay was used as an *in vitro* model to study the epithelial repair potential of BM‐MSC‐EVs (1 × 10^8^) in cultured HCECs. Mitomycin C treatment (10 μg·mL^−1^ for 3 h), before scratch, was used to inhibit the proliferation of HCECs. The epithelial repair was observed and analyzed using microscopic images at different time intervals (0, 12, 24, and 36 h). The area of the wound was calculated using ImageJ and a histogram was plotted with mean areas of wound healing at different time intervals. The total area of the wound at 0 h is expressed as 100%. The magnification bar represents 100 μm. (B) The proliferation assay employed the Ki67 staining and quantification technique to evaluate the proliferative capacity of injured HCECs that were not subjected to Mitomycin C treatment. The assays were performed independently at least three times (*N* = 3) in triplicate and representative images are shown for illustrative purposes. The values are expressed as mean ± SD. *P > 0.05, **P > 0.01, ***P > 0.005, as compared to mock. The magnification bar represents 50 μm.

The role of BM‐MSC‐EVs in corneal epithelial repair was evaluated through the *in vitro* scratch assay [28]. The total area of the scratch at 0 h was considered 100%. Compared with the mock, scratched HCECs incubated with BM‐MSC‐EVs showed a significantly faster rate of wound closure and smaller area of wound at different time points: 12 h (61.9% ± 4.63% vs. 33.58% ± 3.43%), 24 h, (29.99% ± 2.52% vs. 13.04% ± 1.48%), and 36 h (19.8% ± 0.61% vs. 2.43% ± 0.37%) (Fig. 2A, (−) Mitomycin C). Mitomycin C‐treated HCECs showed no significant effect on wound closure. Further, the addition of BM‐MSC‐EVs to Mitomycin C‐treated HCECs could not alter the rate of wound closure, compared to the mock (Fig. 2 A, (+) Mitomycin C).

The proliferative role of BM‐MSC‐EVs in corneal epithelial repair was evaluated through proliferation assay using Ki67 staining. Compared to Mock, the wounded HCECs incubated with BM‐MSC‐EVs displayed a higher number of Ki67‐positive cells and increased mean fluorescence intensity at different time points; 12 h (2433.2 ± 698.4 a.u. vs. 5813 ± 1123.3 a.u.), 24 h (3573.9 ± 753.6 a.u. vs. 9885.1 ± 1690.3 a.u.), and 36 h (7990.6 ± 1293.2 a.u. vs. 12065.9 ± 1051.8 a.u.) (Fig. 2B).

### Regulation of apoptosis

**Fig. 3:**
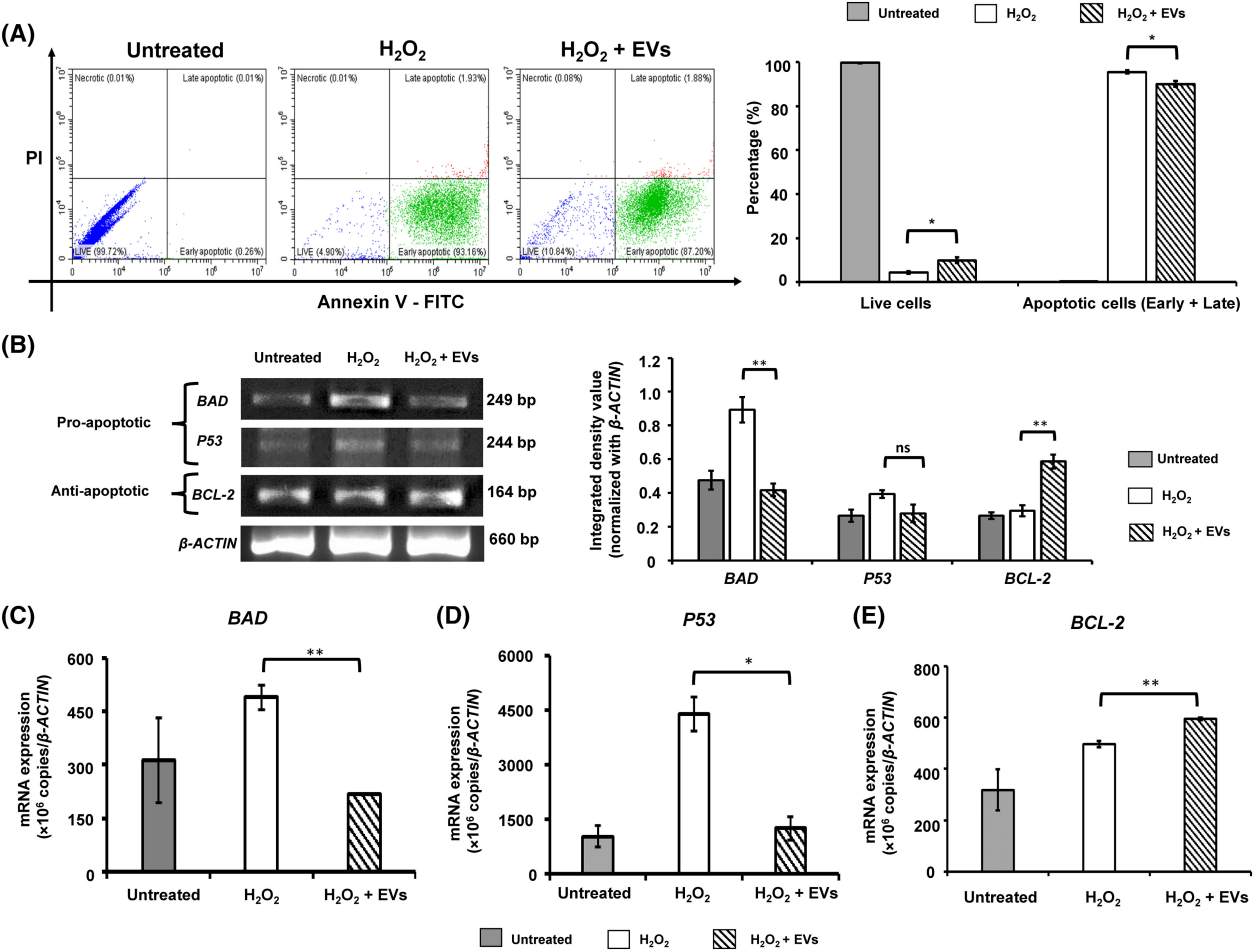
Regulation of apoptosis by BM‐MSC‐EVs. Apoptosis was induced in HCECs using H_2_O_2_ (200 μmol·L^−1^ for 4 h) treatment. The H_2_O_2_‐treated HCECs were incubated with/without BM‐MSC‐EVs (1 × 10^8^) for 24 h. (A) Apoptotic regulation by BM‐MSC‐EVs was observed by flow cytometry‐based double staining with Annexin V‐FITC and Propidium Iodide. The cells were identified as: Live (Annexin V^−^/PI^−^), early (Annexin V^+^/PI^−^), and late (Annexin V^+^/PI^+^) apoptotic. The adjacent bar graph shows the percentage of live and apoptotic (including both early and late) cells. (B) Semi‐quantitative PCR products of pro‐ (*BAD* and *P53*) and anti‐apoptotic (*BCL‐2*) genes were analyzed on 2% agarose gel and representative images are shown. The quantitation was performed using ImageJ and values are expressed as Integrated Density Values (IDV), normalized to *β‐Actin* in the adjacent bar graph. (C–E) show the absolute mRNA expression of *BAD*, *P53*, and *BCL‐2*, respectively, quantified through qPCR*. β‐Actin* was used as an internal control. Experiments were performed in triplicate and independently repeated at least three times (*N* = 3). The values are expressed as mean ± SD. *P > 0.05, **P > 0.01, ns: non‐significant.

Apoptosis was induced in cultured HCECs using treatment with H_2_O_2_ (200 μmol·L^−1^ for 4 h). Flow cytometry analysis suggests that compared with the H_2_O_2_‐treated HCECs alone (95.50 ± 0.58%), HCECs incubated with BM‐MSC‐EVs (H_2_O_2_ + EVs) revealed a significant decrease (90.00 ± 1.30%) in the total apoptotic (early: Annexin V^+^/PI^−^, and late: Annexin V^+^/PI^+^) cells (Fig. 3A).

Compared with H_2_O_2_‐treated HCECs, the incubation with BM‐MSC‐EVs resulted in downregulation of pro‐apoptotic genes: *BAD* (IDV: 0.89 ± 0.07 vs. 0.41 ± 0.03) and *P53* (0.39 ± 0.02 vs. 0.27 ± 0.05), and upregulation of an anti‐apoptotic gene: *BCL‐2* (IDV: 0.29 ± 0.03 vs. 0.58 ± 0.04) (Fig. 3B). Similarly, the absolute quantification of gene expression (×10^6^ copies/*β‐ACTIN*) by qPCR analysis revealed significant downregulation of *BAD* (488.97 ± 35.34 vs. 216.78 ± 1.40, Fig. 3C) and *P53* (4374.52 ± 468.85 vs. 1240.61 ± 327.04, Fig. 3D) and upregulation of *BCL‐2* (497.60 ± 11.91 vs. 595.05 ± 5.32, Fig. 3E).

Further, apoptosis was validated in cultured HCECs through induction with Staurosporine or BAK. Flow cytometry analysis suggests that compared with the Staurosporine‐ or BAK‐treated HCECs alone, HCECs incubated with BM‐MSC‐EVs revealed a significant reduction in PI‐positive (apoptotic) cells (Fig. S1).

### Modulation of CASPASE‐3 expression

**Fig. 4:**
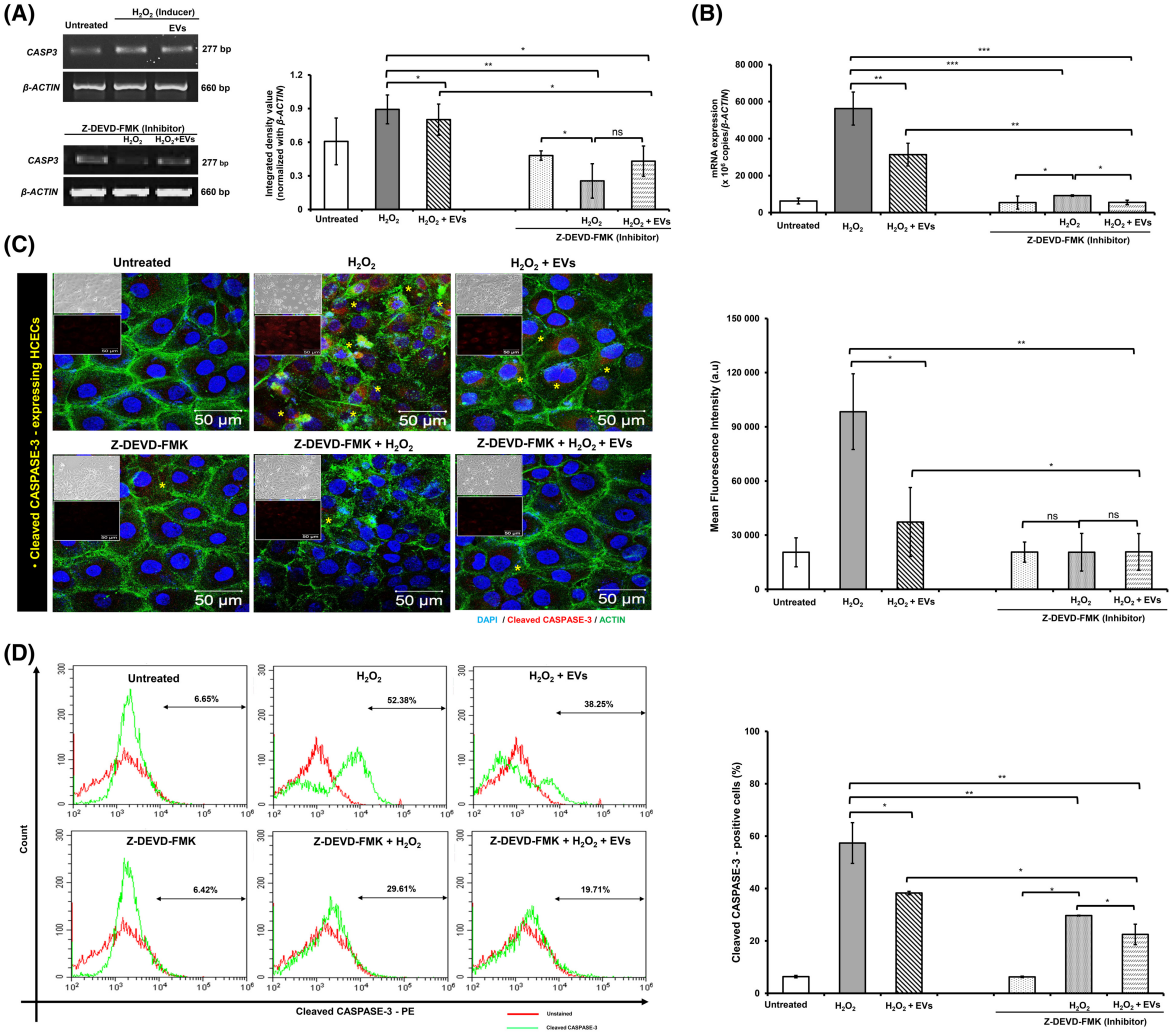
Modulation of CASPASE‐3 (CASP‐3) expression by BM‐MSC‐EVs. Human corneal epithelial cells (HCECs) were subjected to pre‐treatment with or without a Caspase‐3 inhibitor (Z‐DEVD‐FMK, 10 μm for 1 h). Subsequently, cells were exposed to hydrogen peroxide (H_2_O_2_, 200 μmol·L^−1^ for 4 h). Following this apoptosis induction, the HCECs were incubated with or without extracellular vesicles derived from bone marrow mesenchymal stem cells (BM‐MSC‐EVs) at a concentration of 1 × 10^8^ particles·mL^−1^ for 24 h. Untreated HCECs served as the control group. The experimental conditions aimed to investigate the impact of Caspase‐3 inhibition and BM‐MSC‐EV treatment on cellular responses in H_2_O_2_‐treated HCECs. Comparisons were made between untreated and Inhibitor, H_2_O_2_‐treated‐ and H_2_O_2_ + Inhibitor, and H_2_O_2_ + BM‐MSC‐EV‐treated‐HCECs and Inhibitor + H_2_O_2_ + BM‐MSC‐EV‐treated‐HCECs. The mRNA expression of *CASPASE‐3* (*CASP3*) in untreated‐, H_2_O_2_‐treated‐, and H_2_O_2_ + BM‐MSC‐EV‐treated‐HCECs was studied by: (A) semi‐quantitative PCR (representative images) along with its densitometric analysis, and (B) quantitative real‐time PCR. *β‐Actin* was used as a loading/internal control. Protein expression of cleaved CASPASE‐3 was studied by: (C) immunofluorescence (where a set of representative images of phase contrast and single immunostaining with cleaved CASP‐3 antibody are included in the inset of each double‐immunostained (cleaved CASPASE‐3 and β‐Actin) representative image), and (D) flow cytometry. The corresponding quantitative analysis is shown in respective bar graphs. Experiments were performed in triplicate and repeated at least three times (*N* = 3). The values are expressed as mean ± SD. *P > 0.05, **P > 0.01, ***P > 0.005; ns: non‐significant. a.u. represents arbitrary units. The yellow‐colored asterisk (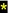) in immunofluorescence images indicates cleaved CASPASE‐3‐expressing HCECs.

In comparison with H_2_O_2_‐treated HCECs, the treatment with BM‐MSC‐EVs (H_2_O_2_ + EVs) resulted in downregulation of *CASPASE‐3 (CASP‐3)* expression in semi‐quantitative (IDV: 0.85 ± 0.23 vs. 0.73 ± 0.009, Fig. 4A) and quantitative real‐time PCR (56198.5 ± 8941.5 vs. 31379.8 ± 6128.9; ×10^6^ copies/*β‐ACTIN*, Fig. 4B).

Interestingly, HCECs treated with Caspase‐3 inhibitor (Z‐DEVD‐FMK) (IDV: 0.48 ± 0.004) resulted in further downregulation of CASPASE‐3 expression in semi‐quantitative PCR. Whereas subsequent treatment with H_2_O_2_ (IDV: 0.25 ± 0.15) or H_2_O_2_ + EVs (IDV: 0.43 ± 0.13) could not change CASPASE‐3 expression (Fig. 4A). The quantitative real‐time PCR also showed the similar results with CASPASE‐3 mRNA expression (Inhibitor, 5435.7 ± 3530.2; Inhibitor + H_2_O_2_, 9196 ± 383.58; Inhibitor + H_2_O_2_ + EVs, 5515.2 ± 1178.7; ×10^6^ copies/β‐ACTIN, Fig. 4B).

Expression of cleaved CASPASE‐3 indicates activation of Caspase‐3‐mediated apoptosis. Compared with H_2_O_2_‐treated HCECs, treatment with BM‐MSC‐EVs (H_2_O_2_ + EVs) suppressed the expression of cleaved CASPASE‐3 indicating reduced apoptosis (Fig. 4C, the yellow asterisk indicates cleaved CASPASE‐3‐expressing cells) and significantly reduced percentage of cleaved CASPASE‐3‐expressing cells (52.38 ± 14.8% vs. 38.25 ± 0.64%, Fig. 4D).

Treatment with Caspase‐3 inhibitor showed significantly reduced expression of cleaved CASPASE‐3 and further treatment with H_2_O_2_ or H_2_O_2_ + EVs could not significantly change its expression. Whereas, compared to Inhibitor (6.22 ± 0.28%), flow cytometry analysis showed a significant increase in the percentage of cleaved CASPASE‐3 upon treatment with H_2_O_2_ (29.71 ± 0.14), however, further incubation with BM‐MSC‐EVs (19.71 ± 3.91) significantly reduced the percentage of cleaved CASPASE‐3 in HCECs.

Combining the results reported in Figs 3 and 4A–D, it appears that the mechanism underlying the observed anti‐apoptotic property of BM‐MSC‐EVs in H_2_O_2_‐treated HCECs in this study involves upregulation of *BCL‐2* and downregulation of *BAD* and *P53*, leading to reduced *CASPASE‐3* (and cleaved CASPASE‐3) expression resulting in inhibition of apoptosis.

## Discussion

Mesenchymal stem cells secrete various bioactive molecules in response to the local environment. EVs isolated from the MSC secretome are currently being employed in the treatment of diverse disorders [35] like cardiovascular diseases, neurodegenerative disorders, bone diseases [36, 37, 38], and dermatological disorders [39]. Multiple studies have demonstrated that EVs derived from human placental MSCs inhibit angiogenesis and inflammation, thereby promoting corneal healing in mice [22, 40, 41]. Furthermore, EVs derived from human umbilical cord MSCs have been shown to promote corneal epithelial cell proliferation and migration via PTEN/PI3K/Akt pathway [42]. Additionally, EVs originating from HCECs have exhibited ultrastructural changes in keratocytes and their transformation into myofibroblasts, aiding in corneal wound healing [43]. Experiments utilizing PKH‐26‐labeled exosomes derived from mouse BM have demonstrated accelerated regeneration of corneal epithelial cells in mice with diabetic keratopathy [44]. In line with these reports, this study reports accelerated corneal epithelial repair by BM‐MSC‐EVs.

Since MSCs are reported to work in a paracrine manner [45, 46], isolated BM‐MSC‐EVs were characterized using TEM, SEM, NTA, western Blotting, and immunofluorescence as per MISEV 2018 guidelines [25]. In line with recently published reports [20], western blotting and immunofluorescence performed in this study confirmed the presence of CD63, CD81, TSG 101, and CD9, and the absence of Calnexin. To assess whether the accelerated corneal epithelial repair by BM‐MSC‐EVs is attributed to the migration or proliferation of HCECs, the proliferation was blocked by Mitomycin C. As a result, we observed very minimal cell migration, indicating that the epithelial repair was predominantly driven by proliferation, consistent with our previous report on umbilical cord‐derived MSCs [28].

This study aimed to investigate the regulatory role of EVs derived from BM‐MSCs on apoptosis in corneal epithelial cells, which play a pivotal role in wound healing. Our results indicate a reduction in the number of apoptotic cells upon treatment with BM‐MSC‐EVs (Fig. 3A). Double staining based on Annexin V and Propidium Iodide (PI) helped in the identification of different cell populations as follows: non‐apoptotic/live cells (Annexin V^−^/PI^−^), apoptotic cells (early apoptotic: Annexin V^+^/PI^−^, late apoptotic: Annexin V^+^/PI^+^), and necrotic cells (Annexin V^−^/PI^+^). BM‐MSC‐EVs suppressed apoptosis and protected epithelial cell loss in the alkali burn mouse model [15] and are reported to show anti‐apoptotic properties in colitis [47], osteoarthritis [48], lung [49], retinal ischemia [50], and corneal endothelium [51]. Furthermore, EVs derived from bone marrow, adipose, and umbilical cord reduced myocardial apoptosis and facilitated angiogenesis [52]. However, to the best of our knowledge, this is the first report indicating the anti‐apoptotic potential of BM‐MSC‐EVs in H_2_O_2_‐induced apoptosis in HCECs.

In this study, we found that the treatment with BM‐MSC‐EVs led to downregulation of pro‐apoptotic *BAD* and upregulation of anti‐apoptotic *BCL‐2*, resulting in the intrinsic apoptotic pathway regulation by Caspase‐3, which is a critical regulator of apoptosis. The function of Caspase‐3 is to cleave and activate Caspases‐6, ‐7, and ‐9 to disintegrate the apoptotic cells before removal; following which the Caspase‐3 is cleaved [53]. The sequential cleaving and activation of these candidates play a critical role in the execution of apoptosis [54]. In this study, most of the H_2_O_2_‐treated HCECs were detected in the early apoptotic‐phase (Annexin V^+^/PI^−^, Fig. 3A) which can be correlated with the cleaved Caspase‐3 expression in immunofluorescence/flow cytometry (Fig. 4C,D) as the early stages of apoptosis involve appearance of active (cleaved) Caspase‐3 in the cytoplasm [55]. The anti‐apoptotic potential of BM‐MSC‐EVs in this study resulted in reduced apoptosis thereby increasing the percentage of live cells and is associated with modulations in the expression of pro‐ and anti‐apoptotic genes. Our findings suggest that the treatment of BM‐MSC‐EVs in H_2_O_2_‐induced HCECs (labeled as H_2_O_2_ + EVs) resulted in upregulation of anti‐apoptotic *BCL‐2*, and downregulation of pro‐apoptotic *BAD* and *P53* expression, leading to downregulation of *Caspase‐3* (*CASP‐3*) expression, resulting in reduced apoptosis. Thus, our results suggest that suppression of Caspase‐3‐mediated apoptosis is one of the key mechanisms underlying anti‐apoptotic potential of BM‐MSC‐EVs in cultured HCECs. These results shed light on the role of BM‐MSC‐EVs in conferring protective effects against apoptosis in corneal epithelial cells.

In line with our results *in vitro*, BM‐MSC‐EVs have been reported to possess anti‐apoptotic properties and promote corneal wound repair by modulating cell death, inflammation, and angiogenesis in murine model of alkali‐burn‐induced corneal damage. These EVs influence the proliferation of corneal cells and lead to a higher abundance of corneal epithelial cells, thus contributing to faster recovery after corneal damage [20]. However, the underlying mechanism of apoptosis is inadequately described. On the other hand, a few studies have reported the pro‐apoptotic behavior of BM‐MSC‐EVs. The latter have been found to partially induce leukemic cell apoptosis through activation of intrinsic and extrinsic apoptosis pathways in an acute promyelocytic leukemia cell line NB4 by significantly increasing the expression of pro‐apoptotic genes BID and BAX, while decreasing the expression of the anti‐apoptotic gene BCL2 [56].

Extracellular vesicles have been shown to have a wide range of therapeutic applications, including in the treatment of cancer, cardiovascular diseases, and neurological disorders. For example, EVs derived from MSCs have been shown to have anti‐inflammatory and immunomodulatory effects, making them a promising therapeutic option for autoimmune diseases [52, 57, 58]. Additionally, EVs have been investigated as a potential drug delivery system [59] as they can be engineered to target specific cells and tissues. Further, EVs derived from various cell types, including MSCs, retinal pigment epithelium, and endothelial cells have therapeutic potential in ocular disorders, such as corneal injury and diabetic retinopathy [60].

Mesenchymal stem cell‐derived extracellular vesicles inherit the immunomodulatory and regenerative properties of MSCs. They contain numerous bioactive molecules from MSCs, such as enzymes (e.g., Nitric oxide synthase), cytokines (e.g., TNF‐α, IFN‐γ, IL‐10), chemokines (e.g., CCL‐17 and CCL‐24), growth factors (e.g., HGF, TGF‐β), mRNA, and microRNAs (e.g., miR497‐5p) [61] – prevent apoptosis in cardiomyocytes induced by sepsis, miR‐126 [62] – diminishes apoptosis in lung endothelial cells by activating PI3K/Akt signaling pathway, similarly many other miRNAs like miR‐21, miR191, miR‐17, miR‐181, and miR‐490‐3p in MSC‐EVs facilitate the suppression of apoptosis (reviewed in [63, 64]). The release of miR‐21 by HUMSC‐sEVs is a critical factor in enhancing the migration and proliferation of HCECs by suppressing PTEN [42]. A study compared ADSC‐EVs vs BM‐MSC‐EVs and reported marked effects of BM‐MSC‐EVs on cell adhesion and metabolic processes, reepithelialization, and proliferation; presumably due to the absence of miRNAs involved in angiogenesis, such as HIF‐1 signaling pathway associated miRNAs as well as miR‐210 and miR378 in BM‐MSC‐EVs, compared with ADSC‐EVs [65]. Additionally, the EVs have been shown to contain several corneal repair‐inducing mediators, such as keratinocyte growth factor, TGF‐β, PDGF, bFGF, EGF, and HGF, which play a crucial role in promoting corneal wound healing and repair [66].

While a healthy cornea is avascular, we acknowledge the fact that healing of the injured cornea may involve corneal angiogenesis/neoangiogenesis in an *in vivo* setting. However, since this study specifically focuses on the role of BM‐MSC‐EVs in corneal epithelial repair *in vitro* (which involves the pure culture of HCECs and no surrounding vascular cells), the study of angiogenesis‐related factors including VEGF is beyond the scope of this investigation. Further, while we acknowledge the importance of examining microRNAs and/or mitochondrial organelle in MSC‐EVs, and the fact that MSC‐EVs have been reported to regulate apoptosis‐related signaling pathways by transporting RNAs (e.g., mRNAs, miRNAs, lncRNAs, and other non‐coding RNAs) [63], the role of BM‐MSC‐EV‐associated RNAs and mitochondrial organelle in regulation of apoptosis is beyond the scope of this investigation. However, our future efforts will be directed towards in‐depth characterization and analysis of cargo of BM‐MSC‐EVs and understanding the role of extracellular RNAs in Caspase‐3‐mediated regulation of apoptosis.

A report by Ming Wai Poon *et al*., [67] explores the role of RAP1 (Telomeric Repeat Binding Factor 2, Interacting Protein) and suggests that deficiency of RAP1 facilitates corneal recovery after injury, while the inhibition of RAP1 may lead to the design of specific inhibitors of NFκB in the treatment of corneal injury. However, RAP‐1 is predominantly associated with large EVs (> 300 nm) [68], while BM‐MSC‐EVs used in this study belong to the small EVs (< 200 nm).

Recent studies on iPSC‐derived MSCs have shown that the iPSC‐MSCs (iMSCs) exhibit strong immunomodulatory properties and have been used in clinical trials for the treatment of refractory graft‐versus‐host disease (GVHD), highlighting their potential as a reliable and consistent resource for clinical applications [52, 69]. Additionally, iPSC‐MSC‐EVs (iMSC‐EVs) have been proposed as a promising alternative to reduce batch‐to‐batch variations and to enhance the quality control of MSC products [70]. The scalability and consistency of iPSC‐MSC production, along with the use of good manufacturing practices (GMP)‐grade iPSC‐MSCs, have been emphasized as key factors in ensuring the safety and efficacy of iPSC‐MSC products [71]. Instead, the tumorigenic potential, low engraftment, and cell retention restricts the therapeutic application of iPSCs [72]. Further, differentiation of iPSCs into MSCs needs caution and optimized protocols to confirm that not even a single iPSC has been left undifferentiated, else the cell product can become tumorigenic [73]. Moreover, the development of iPSC‐based therapies requires higher production costs and is labor‐intensive.

This study is limited in scope as it lacks: (a) *in vivo* implications of BM‐MSC‐EVs in animal models of corneal injury, (b) identification of key components of BM‐MSC‐EVs involved in their anti‐apoptotic and corneal epithelial repair potentials, (c) biological replicates as the EVs have been derived from a single batch of commercially procured bone marrow MSCs, and (d) involvement of the corneal stroma in corneal wound healing. Our future studies are expected to address these limitations.

## Conclusions

BM‐MSC‐EVs accelerate corneal epithelial repair via enhanced cell proliferation in corneal injury and suppress CASPASE‐3 mediated apoptosis *in vitro*. These EVs appear to have the potential to be used as a new approach for treating injury‐induced corneal epithelial defects (Video 1).

**Video 1:** BM‐MSC‐EVs in corneal epithelial repair and apoptosis.

## Conflict of interest

The authors declare no conflict of interest.

## Author contributions

VT performed the experiments, analyzed the data, and wrote the manuscript. SM helped in proofreading the manuscript and data analysis. SB helped in editing the manuscript and provided valuable suggestions. SS designed the study, planned the experiments, supervised the study, analyzed the data, and wrote the manuscript.

## Supporting information


**Fig. S1.** The anti‐apoptotic potential of BM‐MSC‐EVs in HCECs.

**Table S1.** List of primers used for gene expression analysis.

## Data Availability

The data that support the findings of this study are available from the corresponding author Dr Sachin Shukla [sachin@lvpei.org] upon reasonable request.
