# Supplementary material for: Bone marrow mesenchymal stem cell‐derived extracellular vesicles promote corneal epithelial repair and suppress apoptosis via modulation of Caspase‐3 in vitro
Source: FEBS Open Bio. 2024 Apr 29;14(6):968–82. doi: 10.1002/2211-5463.13804 (PMC11494918; doi:10.1002/2211-5463.13804)
Supplement: Supplementary file 1 — Fig. S1. The anti‐apoptotic potential of BM‐MSC‐EVs in HCECs. [file FEB4-14-968-s002.pdf]

Figure S1

Tati V. *et al*

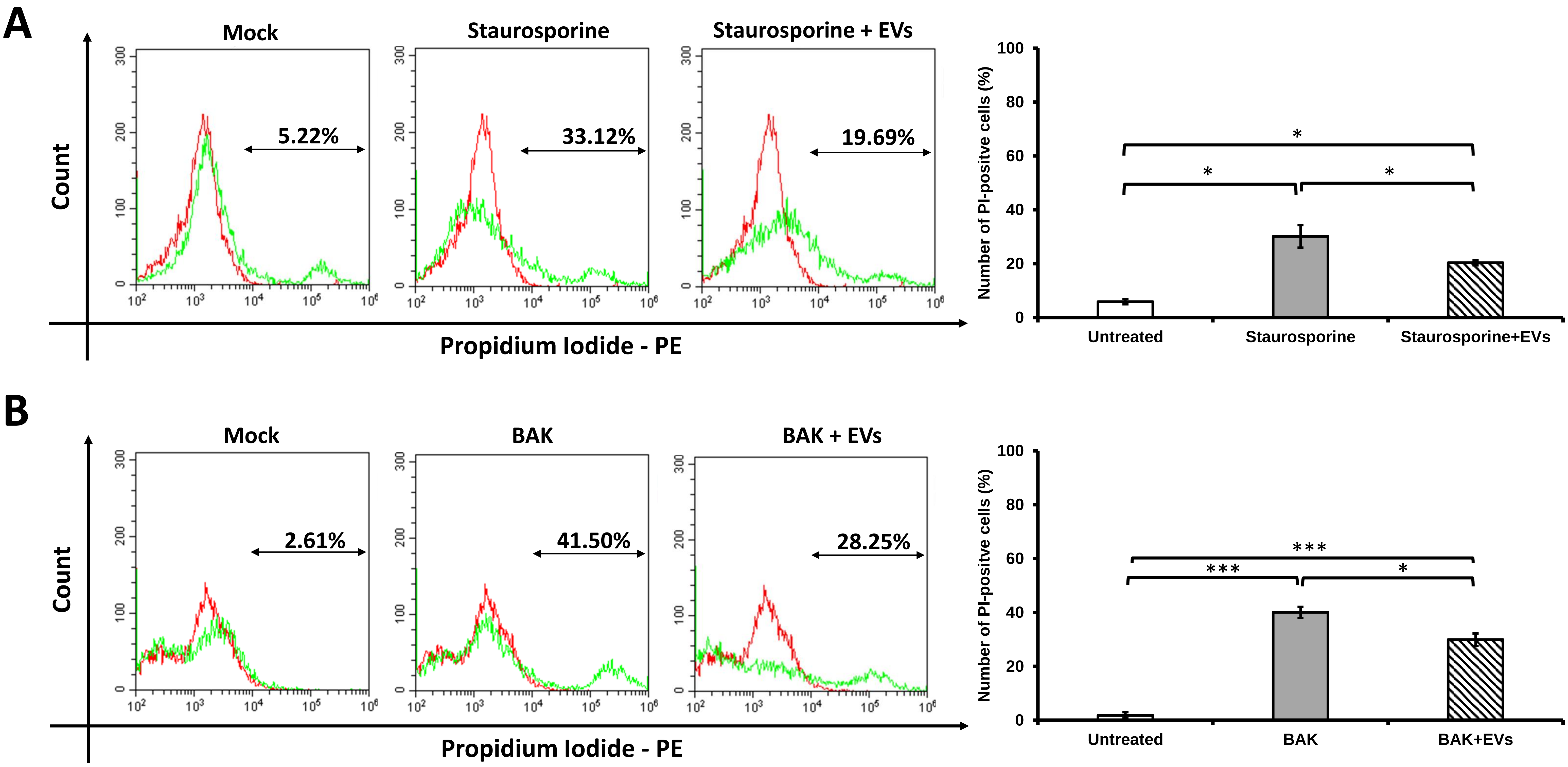

Figure S1. The anti-apoptotic potential of BM-MSC-EVs in HCECs. Apoptosis was induced in HCECs using **(A)** Staurosporine (STS) (0.25µM for 3 hours) and **(B)** Benzalkonium chloride (BAK) (0.005% for 15 minutes) treatment. The Staurosporine or BAK-treated HCECs were incubated with/without BM-MSC-EVs ($1x10^8$ /ml) for 24 hours. The anti-apoptotic potential of BM-MSC-EVs was observed by flow cytometry-based Propidium Iodide (PI) staining. The PI-positive cell percentage was plotted as a histogram in an adjacent bar graph. The values are expressed as mean ± SD. *p>0.05, ***p>0.005.
