# Supplementary material for: Bone marrow mesenchymal stem cell‐derived extracellular vesicles promote corneal epithelial repair and suppress apoptosis via modulation of Caspase‐3 in vitro
Source: FEBS Open Bio. 2024 Apr 29;14(6):968–82. doi: 10.1002/2211-5463.13804 (PMC11494918; doi:10.1002/2211-5463.13804)
Supplement: Supplementary file 2 — Table S1. List of primers used for gene expression analysis. [file FEB4-14-968-s001.pdf]

Table S1: List of primers used for gene expression analysis.

Primer	Sequence	Amplicon size
BAD	Forward: 5’ CCCAGAGTTTGAGCCGAGTG 3’<br>Reverse: 5’ CCCATCCCTTCGTCGTCCT 3’	249 bp
BCL-2	Forward: 5’ GAACTGGGGGAGGATTGTGG 3’<br>Reverse: 5’ CATCCCAGCCTCCGTTATCC	164 bp
P53	Forward: 5’ CCCAGAAGGACTGCACCAAT 3’<br>Reverse: 5’ GTTCCTCCCTGCCCTTTCTC 3’	244 bp
β-ACTIN	Forward: 5’ TGACGGGGTCACCCACACTGTGCCCATCTA 3’<br>Reverse: 5’ CTAGAAGCATTTGCGGTGGACGATGGAGGG 3’	661 bp
CASP3 [74]	Forward: 5′-CCT AGC GGA TGG GTG CTA TT-3′<br>Reverse: 5′-CTG AGG TTT GCT GCA TCG AC-3′	277bp
